# Raw potato starch diet supplement in weaned pigs could reduce *Salmonella* Typhimurium infection by altering microbiome composition and improving immune status

**DOI:** 10.3389/fvets.2023.1183400

**Published:** 2023-05-23

**Authors:** Seung-Won Yi, Han Gyu Lee, Eunju Kim, Young-Hun Jung, Eun-Yeong Bok, Ara Cho, Yoon Jung Do, Tai-Young Hur, Sang-Ik Oh

**Affiliations:** ^1^Division of Animal Diseases and Health, National Institute of Animal Science, Rural Development Administration, Wanju-gun, Jeollabuk-do, Republic of Korea; ^2^Laboratory of Veterinary Pathology, College of Veterinary Medicine, Jeonbuk National University, Iksan, Jeollabuk-do, Republic of Korea

**Keywords:** diarrhea, microbiome, raw potato starch, resistant starch, *Salmonella* Typhimurium, weaned pig, cytokine, transcriptome

## Abstract

**Backgorund:**

*Salmonella enterica* serovar Typhimurium (ST) is one of the causative agents of gastroenteritis in pigs. Pigs fed a diet supplemented with raw potato starch (RPS) have improved gut health by the alteration of the microbiota composition and production of short-chain fatty acids (SCFAs). This study aimed to evaluate the effects of RPS supplementation in reducing infection severity and fecal shedding in ST-infected pigs.

**Methods:**

The weaned experimental pigs were divided into two groups: CON (*n* = 6) fed a corn/soybean-based diet and TRT (*n* = 6) supplemented with 5% RPS. After 21 d, the pigs were inoculated with ST, and their body weight, clinical signs, and fecal shedding of ST were monitored for 14 d. At 14 d post-inoculation (dpi), the jejunum, cecum, ileum, and colon tissues were collected from euthanized pigs, and histopathological lesions and cytokine gene expression were compared. Additionally, blood samples at 2 dpi were analyzed for gene ontology enrichment. Moreover, the gutmicrobiome was analyzed using 16S rRNA metagenomic sequencing, and the SCFA concentration was measured using gas chromatography.

**Results:**

The average daily weight gain was significantly higher in TRT than in CON during the ST infection period; however, histopathological lesion scores were significantly lower in TRT than in CON. The relative abundance of nine genera of butyrate- and acetate-producing bacteria significantly increased in TRT compared with that of only two acetate-producing bacteria in CON. Among the genes involved in the immune response, IL-18 expression level was significantly lower in the jejunum and colon in TRT than in CON. Furthermore, *Reg3*γ expression was significantly different in the cecum and colon of both groups.

**Conclusion:**

The diet supplemented with RPS in weaned pigs could result in predominance of butyrate- and acetate-producing bacteria, reducing the severity of ST infection by improving the immune status.

## 1. Introduction

*Salmonella* is an important foodborne pathogen that can cause human and animal infections (1). Among the various serovars of *Salmonella*, non-typhoidal *Salmonella* infections are a major public health problem worldwide (1). Pigs are an important non-typhoidal *Salmonella* infection source for humans, especially *Salmonella enterica* serovar Typhimurium (ST) (2). Intestinal inflammation caused by *Salmonella* infection can disrupt commensal microbiota and gut barriers, resulting in the bacteria colonizing the tissues of the host intestine (3, 4), leading to diarrhea, fibrinonecrotic enterocolitis, and dehydration in pigs (5).

Healthy gut microbiota can reduce the severity of *Salmonella* infection; therefore, a feed supplement diet that supports beneficial microbial populations is a potential on-farm strategy to control *Salmonella* infection in pigs (6, 7). Resistant starch (RS) is an important source of microbiota-accessible carbohydrates because it is digested in the large rather than the small intestines (8). RS feeding can increase short-chain fatty acids (SCFAs) in the intestinal tract, resulting in improved barrier functions, enhanced tolerance to commensal organisms, and reduced inflammation in the gut tissue (9, 10). Raw potato starch (RPS) is a common ingredient of RS that improves fermentation in the digestive tract and increases pro- and anti-inflammatory cytokine levels (11). Recently, studies have shown that RPS feeding could increase gene expression related to the cecal barrier function and improve the mucosal immune system in animals (8, 12).

Our previous study revealed that RPS consumption could promote the growth of beneficial microbes, promoting SCFA production in weaned pigs (13). Therefore, we hypothesized that feeding RPS as a supplement could reduce ST infection severity and fecal shedding in weaned pigs. This study aimed to determine the effect of feeding RPS in reducing ST infection severity in weaned pigs by comparing ST shedding and colonization, histopathological lesions, microbiota composition, and immunological responses in ST-inoculated pigs fed RS and non-RS diets. The findings could provide potential methods for preventing ST infection in pigs by altering microbiome composition and improving immune responses.

## 2. Materials and methods

### 2.1. Animal ethics

All experiments were approved by the Animal Ethics Committee of the National Institute of Animal Science, Republic of Korea (Approval No. NIAS 2021-503).

### 2.2. Animals, experimental diets, and *Salmonella* Typhimurium inoculation

Twelve castrated male piglets (Landrace × Yorkshire, aged 25 d) were obtained from the same herd in a commercial farm. The average weight of the pigs was 5.00 ± 0.8 kg. All pigs were carefully monitored daily for 3 d before the diet experiment. During the adaptation period, all pigs were confirmed to be sero-negative for foot-and-mouth disease, porcine respiratory and reproductive syndrome, classical swine fever, *Mycoplasma* spp. infection, and *Salmonella* spp. infection. In addition, *Salmonella* spp. and *Escherichia coli* were not detected in the fecal samples of the experimental pigs. Piglets (aged 28 d) were randomly divided into two groups: the treatment (TRT, *n* = 6) and negative control (CON, *n* = 6) groups. The CON diet was formulated according to the nutritional requirements suggested by the Korean feeding standard for pigs, and the TRT pigs were fed the CON diet supplemented with 5% RPS for 21 d (Table 1). After 21 d, the TRT pigs' diet (aged 49 d) was changed to the CON diet until the end of the experiment. Subsequently, all experimental pigs aged 49 d were orally inoculated with 1 × 10^8^ colony forming units of ST LT2 strain (ATCC 19585). The ST-infected pigs were then euthanized 14 d after bacterial inoculation and immediately necropsied to collect the tissue samples.

**Table 1:** Ingredients and chemical composition of the experimental diet.

**Items**	**TRT (%)**	**CON (%)**
**Ingredients**		
Corn	68.24	73.74
Soybean meal 44%	23.25	22.20
Soybean oil	0.33	0.86
L-Lysine-HCl	0.15	0.17
Dicalcium phosphate	1.15	1.15
Limestone	0.88	0.88
Vitamin-mineral premix^a^	0.50	0.50
NaCl	0.50	0.50
Raw potato starch	5.00	-
**Calculated composition**		
Metabolizable energy (kcal/kg)	3,300	3,300
Crude protein	16.00	16.0
Lysine	0.95	0.95
Methionine	0.26	0.26
Calcium	0.66	0.66
Total protein	0.56	0.56

a Values supplied per kilogram premix feed concentrations: Vitamin A 5,000,000 IU; vitamin E, 1,000 mg; Vitamin B_1_, 150 mg; Vitamin B_2_, 300 mg; Vitamin B_12_, 1,500 mg; niacin amide, 1,500 mg; DL-calcium pantothenate, 1,000 mg; folic acid, 200 mg; Vitamin H, 10 mg; choline chloride, 2,000 mg; min 3,800 mg; zinc, 1,500 mg; iron, 4,000 mg; Cu, 500 mg; I, 250 mg; Co, 100 mg; Mg, 200 mg.

TRT, piglets fed the control formula diet supplemented with 5% raw potato starch; CON, piglets fed the corn/soybean control formula diet.

### 2.3. Sampling and evaluating *Salmonella* shedding

Fecal samples were collected at 0 and 21 d post-feeding (dpf) and 2, 5, 8, and 11 d post-ST inoculation (dpi). The fecal and intestinal tissues (jejunum, ileum, colon, and cecum) were collected from euthanized pigs at 14 dpi. The Rappaport–Vassiliadis R10 broth (BD, Sparks, MD, USA) containing the fecal and tissue samples (1 g) was incubated immediately at 42°C for 24 h, and one loop of the RV culture was streaked onto CHROMagar *Salmonella* Plus (CHROMagar, Paris, France). Lastly, the mauve colonies were identified as ST by polymerase chain reaction (PCR) using the *AccuPower Salmonella* spp. 3-Plex PCR Kit (Bioneer, Daejeon, Korea).

### 2.4. Histopathology and immunohistochemistry

Tissue samples from the jejunum, ileum, cecum, and colon of necropsied pigs (at 14 dpi) were fixed in 10% neutral-buffered formalin and embedded in paraffin wax. Subsequently, 4-μm-thick sectioned tissues were stained with hematoxylin and eosin using a standard laboratory protocol and immunohistochemically stained with anti-*Salmonella* Typhimurium (BS-4801R; Thermo Fisher Scientific, Rockford, IL, USA). Lastly, the histopathological lesions were scored (from 0 to 5) using previously described parameters, including villus shortening and erosion, presence and concentration of ST, and inflammatory cell infiltration (14).

### 2.5. Microbial community analysis

DNA was extracted using the DNeasy PowerSoil Kit (Qiagen, Hilden, Germany) following the manufacturer's instructions. Amplicons of the V3–V4 region were generated and sequenced following the Illumina 16S metagenomic sequencing library preparation protocol. Subsequently, paired-end sequencing of the amplicon was performed using the MiSeq platform (Illumina, San Diego, CA, USA). Afterward, bioinformatics analysis was performed as described in our previous study (13). Lastly, sequencing data were arranged according to the two experimental groups (TRT and CON) for analytical purposes.

### 2.6. Measurement of SCFA concentrations

Acetate, butyrate, and propionate were selected based on their specific differences reported in our previous study (13). Fecal concentrations of SCFAs were determined using an Agilent 6890 series gas chromatograph (Agilent Technologies, Santa Clara, CA, USA) coupled with mass spectrometry (13).

### 2.7. Quantification of cytokines in gastrointestinal tissue

The jejunum, cecum, and colon tissues from pigs at 14 dpi were analyzed for cytokine quantification. First, total RNA was isolated using the RNeasy Mini Kit (Qiagen, Hilden, Germany) and reverse-transcribed into cDNA using a High-Capacity cDNA Synthesis Kit (Applied Biosystems, Foster City, CA, USA) following the manufacturer's instructions. Additionally, the relative expression of seven genes was quantified by reverse transcription (RT) PCR (RT-PCR), including four genes related to gut barrier function [claudin (CLDN), occludin (OCLN), zonula occludens-1 (ZO-1)], and regenerating islet-derived protein 3-gamma (Reg3γ), and three genes related to the immune response against *Salmonella* infection, including interleukin (IL)-10, IL-17A, and IL-18. Notably, RT-PCR was performed using the ABI 7500 Real-Time PCR System (Applied Biosystems) under the following conditions: 10 min at 95°C, 40 cycles at 95°C for 15 s, the annealing temperature of each primer for 30 min, and 72°C for 15 s. The primers and annealing temperatures are listed in Table 2. Lastly, the expression fold change was determined using the 2^−ΔΔ*Ct*^ method with the beta-actin gene as the endogenous reference gene to normalize the level of target gene expression.

**Table 2:** The primer information and PCR condition for quantitative real-time polymer chain reaction.

**Primer**	**Target**	**Sequence (5^′^ → 3^′^)**	**Size (bp)**	**Annealing temp (°C)**	**Reference**
IL-10-F	Interleukin 10	GCCTTCGGCCCAGTGAA	101 bp	62°C	(15)
IL-10-R		AGAGACCCGGTCAGCAACAA			
IL-17A-F	Interleukin 17A	CCCTGTCACTGCTGCTTCTG	57 bp	62°C	(16)
IL-17A-R		TCATGATTCCCGCCTTCAC			
IL-18-F	Interleukin 18	ACGACCAAGTCCTTTTCATTAACC	85 bp	63.6°C	(17)
IL-18-R		TGAGGTGCATTATCTGAACAGTCA			
ZO-1-F	Zonula occludin-1	GGCTCTTGGCTTGCTATTCG	98 bp	62.0°C	
ZO-1-R		TGGACACTGGCTAACTGCTCA			
OCLN-F	Occludin	CCAACGGGAAAGTGAACGAG	149 bp	63.0°C	
OCLN-R		CGCCTCCAAGTTACCACTGC			
CLDN1-F	Claudin-1	AACCCGTGCCTTGATGGTAA	127 bp	62.6°C	
CLDN1-R		AATGACAGCCATCCGCATCT			
REG3γ-F	Regenerating islet-derived 3 gamma	TGTCTCAGGTCCAAGGTGAAGA	102 bp	62.2°C	
REG3γ-R		ACAAGGCATAGCAGTAGGAAGCA			
DefB-F	Beta defensin 1	CTCTGCTTGCTGCTGCTGAC	188 bp	62.2°C	
DefB-R		CACTTGGCCTTGCCACTGTA			
ACTB-F	Beta-actin	CAAATGCTTCTAGGCGGACTGT	75 bp	-	(15)
ACTB-R		TCTCATTTTCTGCGCAAGTTAGG			

### 2.8. RNA sequencing data and differentially expressed gene (DEG) analysis

Total RNA was obtained from all experimental pigs at 2 dpi using a Tempus Blood RNA Tube (Applied Biosystems, Seoul, Korea). To produce the transcriptome, TNT Research Corporation Limited (Anyang, Korea) conducted RNA and cDNA library construction and RNA sequencing as previously described (18). Lastly, the DEGs in TRT and CON groups' blood were analyzed based on the expression level of each transcript, as previously described (18).

### 2.9. Statistical analysis

The Kruskal–Wallis and unpaired Wilcoxon rank-sum tests were used for comparing the alpha diversities of the fecal microbiome composition in the TRT and CON groups at 14 dpi and three time points (0, 21 dpf, and 14 dpi), respectively. All statistical analyses adopted a *P*-value of 0.05 as the cut-off value and a linear discriminant analysis (LDA) score of 2.0 using the QIIME software version 2.0. Moreover, beta diversity was visualized by principal coordinate analysis (PCoA) matrix using Bray–Curtis distance and QIIME software version 2.0. Additionally, the LDA effect size (LEfSe) was used to determine the specific effect on the relative abundance of taxa in the RS and non-RS groups. Taxa with a significant difference (*P* < 0.05) between both groups were subjected to LEfSe analysis, and those with LDA score > 2.0 were considered to have been significantly altered after *Salmonella* infection in the TRT group compared with those in the CON group. Lastly, significant changes in average daily gain (ADG), histopathological lesion scores, and SCFA concentrations between the TRT and CON groups were compared by Student's *t*-test using the SPSS software (version 26.0; IBM, Armonk, NY, USA).

## 3. Results

### 3.1. Growth performance and *Salmonella* Typhimurium fecal shedding

**Figure 1:**
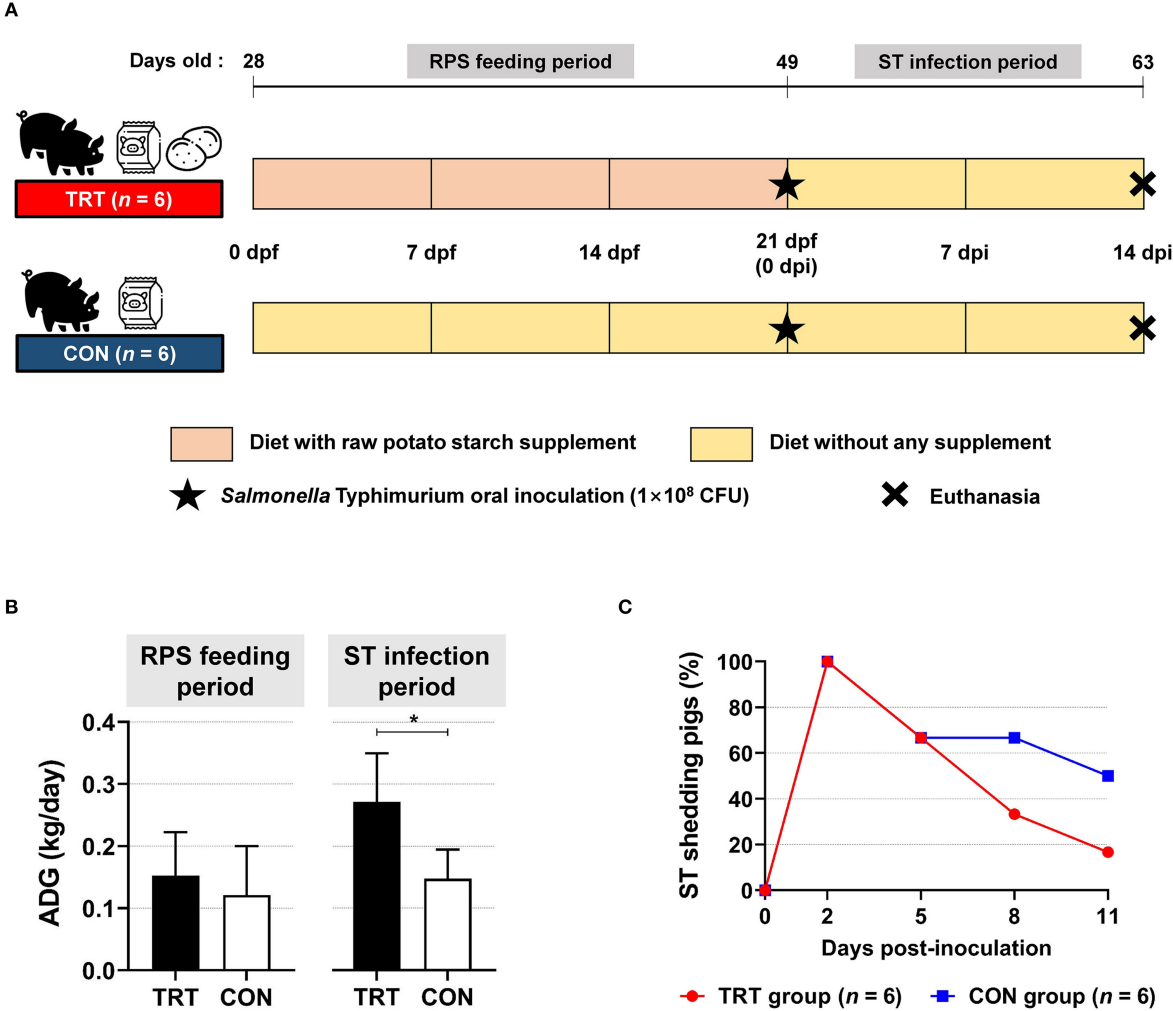
Effect of RPS diet supplement in *Salmonella* Typhimurium (ST)-infected weaned pigs. **(A)** Experimental scheme. **(B)** Average daily gains (ADG) in RS-fed (TRT) and non-RS-fed pigs (CON) during two experimental periods. *P < 0.05. **(C)** Isolation of ST in fecal samples from TRT and CON until 11 d post-inoculation.

We compared the pigs in the TRT group (fed with RPS supplemented diet) with those in the CON group (Figure 1A). The ADG during the feeding period (from 0 to 21 dpf) was 0.15 and 0.12 kg/day in the TRT and CON groups, respectively; however, ADG during ST infection period (from 0 to 14 dpi) in the TRT group (0.27 kg/day) was significantly (*P* = 0.010) higher than that in the CON group (0.15 kg/day) (Figure 1B). Moreover, the TRT group showed marked reductions in ST shedding at 8 and 11 dpi compared with the CON group [8 dpi: 33.3% (TRT) *vs*. 66.7% (CON) and 11 dpi: 16.7% (TRT) *vs*. 50.0% (CON)] (Figure 1C).

### 3.2. Histopathological lesions and *Salmonella* Typhimurium isolation in tissues

**Figure 2:**
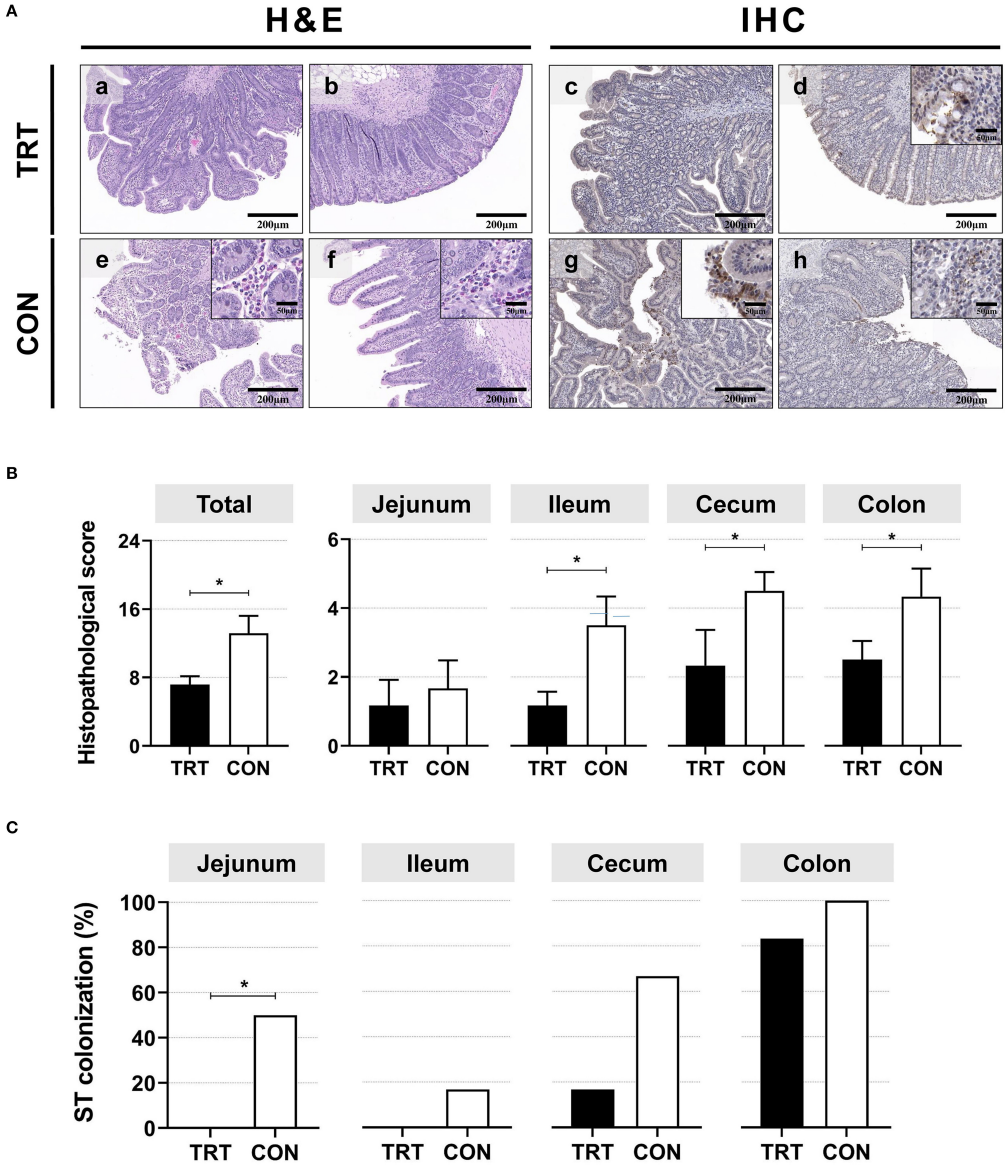
Histopathological lesions in experimental pigs and *Salmonella* Typhimurium (ST) isolation from intestinal organ tissues. **(A)** Representative hematoxylin and eosin and immunohistochemical (anti-ST) staining of the ileum (a, e), cecum (b, f), jejunum (c, g), and colon (d, h). **(B)** Histopathological scores of the intestinal organs (jejunum, ileum, cecum, and colon). *P < 0.05. **(C)** Isolation of ST from the intestinal organs (jejunum, ileum, cecum, and colon). *P < 0.05.

Although histopathological lesions and ST were observed in both groups (Figure 2A), the CON pigs exhibited more severe organismal damage than the TRT pigs, indicated by the total average histopathological lesion scores in the jejunum, ileum, cecum, and colon [TRT (1.8 ± 0.9) vs. CON (3.6 ± 1.3), *P* < 0.001] (Figure 2B). Additionally, the average scores in the TRT pigs' ileum (1.2 ± 0.4), cecum (1.2 ± 0.4), and colon (2.5 ± 0.5) were significantly lower (*P* < 0.001, *P* = 0.001, and *P* < 0.001, respectively) than those in the CON pigs' ileum (3.5 ± 0.8), cecum (4.5 ± 0.5), and colon (4.3 ± 0.7).

For intestinal tissues, the estimated isolation rate of ST from the TRT pigs was lower than that from the CON pigs (Figure 2C). The isolation rate from the TRT pigs' jejunum (0%, zero of six pigs) was significantly (*P* = 0.046) lower than that for the CON pigs (50.0%, three of six pigs). Moreover, ST was detected in the ileum of only one CON pig. Lastly, one TRT pig (16.7%) and four CON pigs (66.7%) harbored ST in the cecum, and the bacteria were isolated from the colon tissue in all pigs, excluding one TRT pig.

### 3.3. Bacterial communities and SCFA concentration

**Figure 3:**
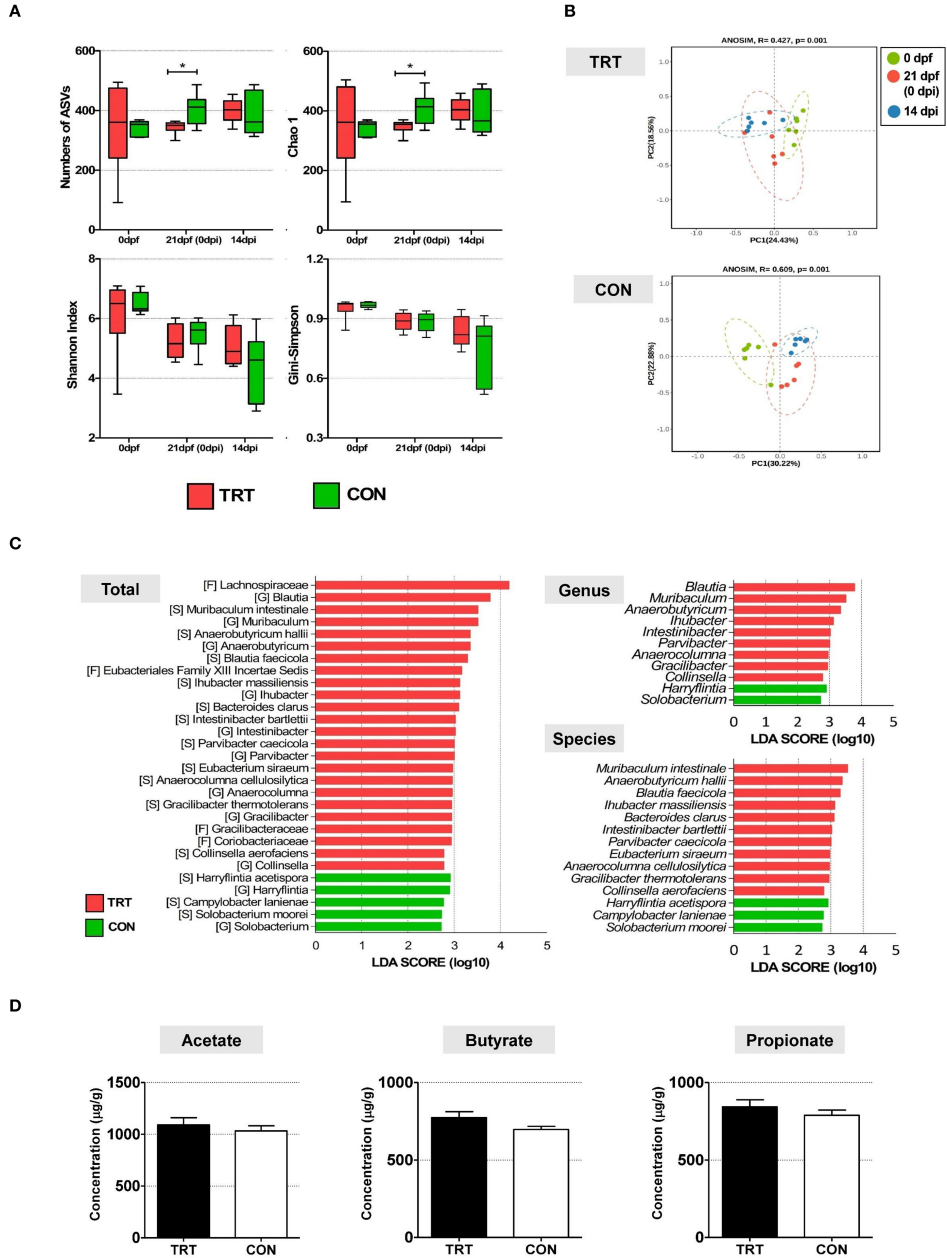
The comparison of alteration of microbiota diversity and composition and SCFA concentration in RPS-fed pigs (TRT) and non-RPS-fed pigs (CON) after *Salmonella* Typhimurium inoculation (at 14 dpi). **(A)** Comparison of alpha diversity of microbiome from feces between TRT and CON. The four indices included the number of ASV, Chao1 richness indices, and Shannon and Gini –Simpson diversity indices. **(B)** PCoA of beta diversity analysis based on the Bray–Curtis dissimilarity matrix. **(C)** LEfSe revealed predicted biological effect sizes of differential taxa in fecal microbiota between TRT and CON. The LDA scores show a significant difference in the abundance and consistency of the detected bacterial taxa at the genus and species levels. **(D)** Concentrations of three SCFAs (acetate, propionate, and butyrate) (μg/g) in TRT and CON fecal samples at 14 dpi. *P < 0.05.

Alpha diversity in fecal microbiota was compared between TRT and CON groups and time points (0, 21 dpf, and 14 dpi) (Figure 3A). Four indices were analyzed, including the number of amplicon sequence variants (ASV), Chao1 richness indices, and Shannon and Gini–Simpson diversity indices. The Kruskal–Wallis test showed no significant differences in the four alpha diversity indices: the number of ASV (*P* = 0.7) and Chao1 richness indices (*P* = 0.7), and Shannon (*P* = 0.59) and Gini–Simpson indices (*P* = 0.59). For the TRT group, the Gini–Simpson index (*P* = 0.016) was significantly higher at 14 dpi than at 0 and 21 dpf. Moreover, the Gini–Simpson (*P* = 0.0015) and Shannon indices (*P* = 0.0017) were significantly lower in the CON group than in the TRT group at 14 dpi. Furthermore, we performed beta diversity analysis to investigate the structure of the bacterial community at 14 dpi; the results are presented as a PCoA ordination plot based on Bray–Curtis distance matrices. Beta diversity differed between the TRT and CON groups at 14 dpi; however, no significant difference was observed between both groups (*P* = 0.529). The bacterial communities during ST infection in the TRT group shifted leftward along the PC1 axis in the opposite direction to those in the CON group (Figure 3B). Moreover, LEfSe analysis showed that 9 bacterial genera and 11 species of fecal microbes were significantly (*P*<*0.05*) increased in the TRT group; however, the abundance of only two genera and three species significantly increased in the CON group (Figure 3C).

The concentrations of three SCFAs (acetate, butyrate, and propionate) were evaluated in all experimental pig fecal samples (14 dpi) to investigate the effect of altered bacterial communities in the TRT and CON groups (Figure 3D). The levels of the three SCFAs were higher in the TRT group than in the CON group. Additionally, the concentrations of acetate, butyrate, and propionate were 1,090.8 ± 170.3 μg/g, 843.3 ± 110.6 μg/g, and 773.6 ± 93.8 μg/g, respectively, in the TRT group, and 1,033.4 ± 120.4 μg/g, 789.3 ± 81.6 μg/g, and 697.3 ± 49.6 μg/g, respectively, in the CON group.

### 3.4. Cytokine expression

**Figure 4:**
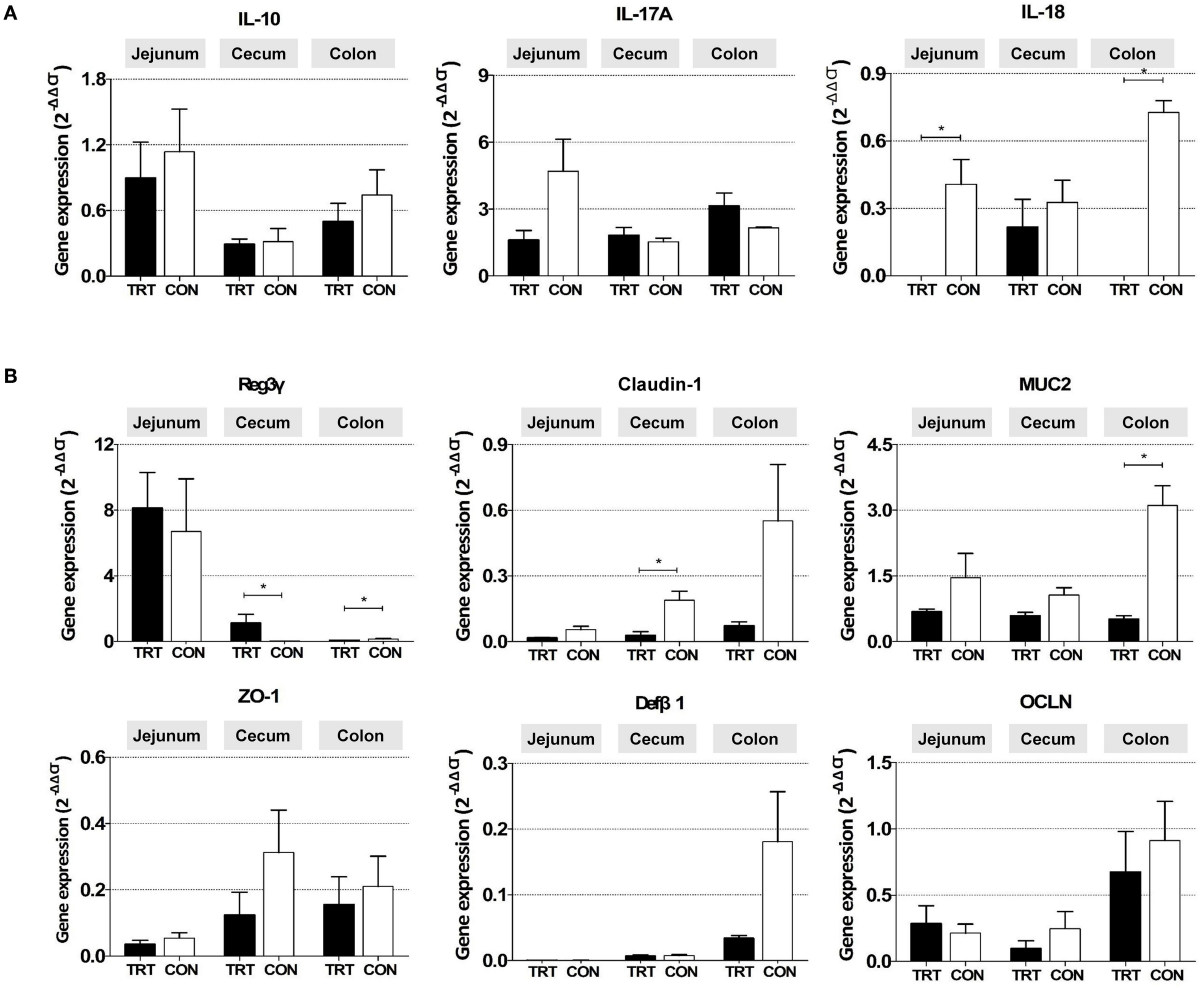
Gene mRNA expression in three intestinal organs (jejunum, cecum, and colon) from RPS-fed (TRT) and non-RPS-fed pigs (CON) after *Salmonella* Typhimurium inoculation (at 14 dpi). **(A)** Genes associated with inflammatory response (*IL-10, IL-17A*, and *IL-18*). **(B)** Genes associated with gut barrier function (*Reg3*γ*, Claudin-1, MUC2, Zo-1, Def*β*1*, and *OCLN*). *P < 0.05.

The relative mRNA expression of the proinflammatory cytokine IL-18 was significantly lower in the jejunum [0.00010 ± 0.00014 (TRT) vs. 0.41 ± 0.11 (CON); *P* = 0.0139] and colon of the TRT group [0.00003 ± 0.00002 (TRT) vs. 0.73 ± 0.05 (CON); *P* = 0.00001] than in those of the CON group (Figure 4A). Additionally, the anti-inflammatory cytokine IL-10 was less expressed in the jejunum, cecum, and colon. Moreover, the expression of the proinflammatory cytokine IL-17A was higher in the TRT group's colon than in the CON group's colon; however, the differences were insignificant due to the large deviations between samples.

Among the genes related to the gut barrier, the expression of the antimicrobial peptide gene *Reg3*γ was significantly higher in the cecum [1.120 ± 0.535 (TRT) *vs*. 0.023 ± 0.004 (CON); *P* = 0.0309] but lower in the colon of the TRT group [0.073 ± 0.012 (TRT) vs. 0.148 ± 0.036 (CON); *P* = 0.0190] than in those of the CON group (Figure 4B). Furthermore, the relative mRNA expression levels of *CLDN-1* in the cecum [0.216 ± 0.124 (TRT) *vs*. 0.327 ± 0.099 (CON); *P* = 0.0254] and *MUC2* in the colon [0.51 ± 0.08 (TRT) vs. 3.11 ± 0.45 (CON); *P* = 0.0095] were significantly lower in the TRT group than in the CON group. However, insignificant difference was observed in *CLDN-1* expression in the jejunum and colon, *MUC2* in the jejunum and cecum, and the other three genes (*ZO-1, OCLN*, and *DefB1*) in the jejunum, cecum, and colon between the two feeding groups.

### 3.5. Transcriptome analysis of the blood sample

**Figure 5:**
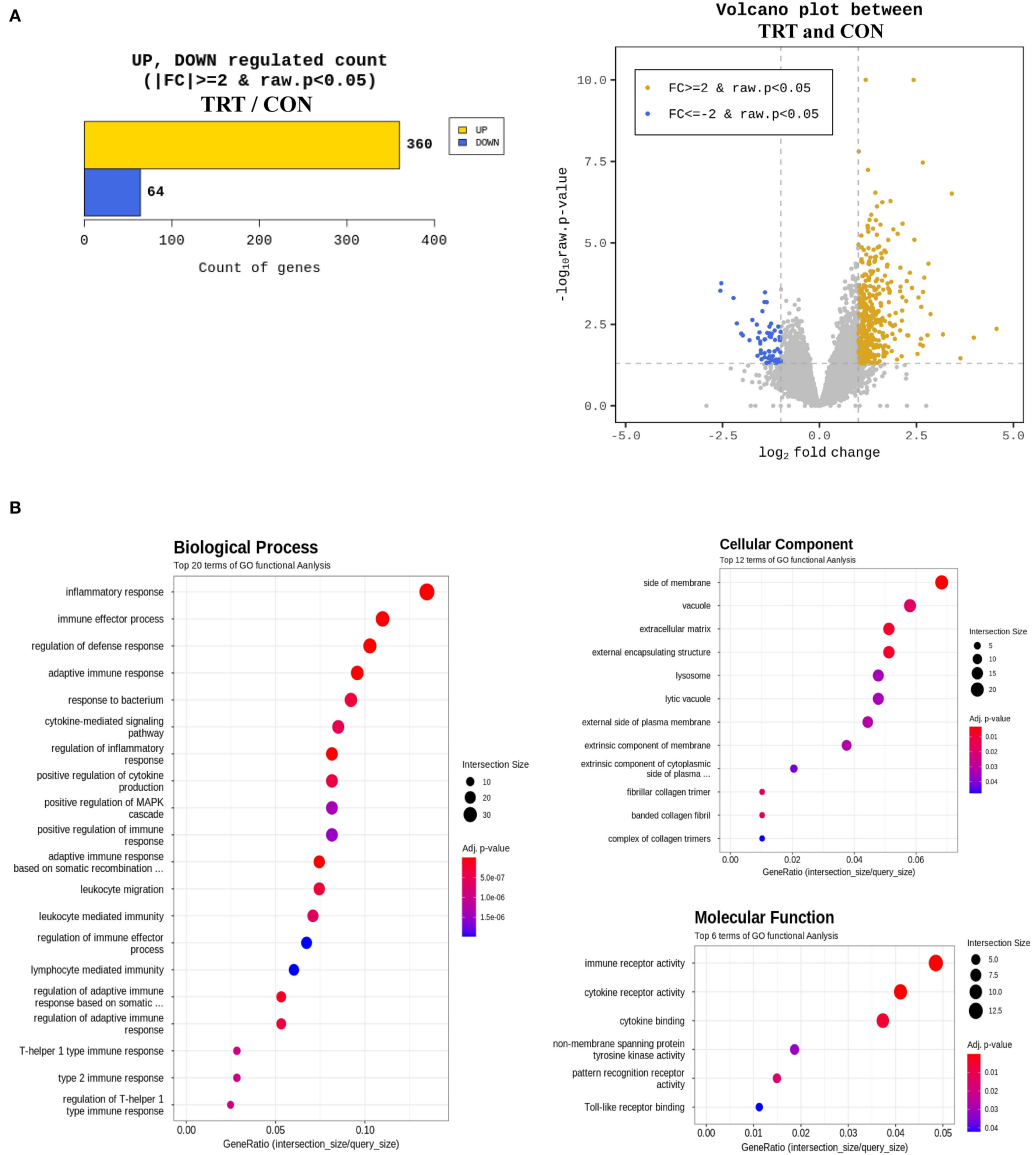
Differentially expressed genes (DEG) and gene ontology (GO) enrichment analysis in blood samples from RPS-fed (TRT) and non-RPS-fed pigs (CON) at 2 d after *Salmonella* Typhimurium inoculation. **(A)** Numbers of up- and down-regulated genes after comparison of normalized values using the DESeq2 package. **(B)** GOTERM_Biological process, GOTERM_Cellular Component, and GOTERM_Molecular Function.

An average of 6.7 Gb raw data for each sample were collected from paired-end transcriptome sequencing using the Illumina NovaSeq 6000 platform. Raw data were subjected to quality control using Trimmomatic (ver. 0.38), and the trimmed data were mapped using HISAT2 ver. 2.1.0 (Bowtie2 aligner). Next, the mapped reads were assembled using the StringTie-e option ver. 1.3.4d. Afterward, the obtained genes were filtered by excluding those with at least one zero count, leaving 12,189 genes for DEG analysis. Overall, 424 genes were considered differentially expressed based on the threshold level (fold change [log_2_] ≥ 2 and *P* < 0.05) (Figure 5A). Lastly, the biological function of 424 DEGs was determined by gene ontology (GO) and Kyoto Encyclopedia of Genes and Genomes pathways using the DAVID 6.8 tool. Figure 5B shows the GO functional analysis of the biological process (top 20), cellular component (top 12), and molecular function (top 6).

## 4. Discussion

*Salmonella* is a major causative agent of diarrhea in pigs, threatening food safety and human health. Local inflammation in *Salmonella*-infected pigs can reportedly cause changes in the gut microbiome, favoring the survival of *Salmonella* (4, 19). We previously revealed that RPS feeding of weaned pigs could improve gut health by maintaining the balance of beneficial bacteria and promoting SCFA production (13). Therefore, we investigated the gut microbiota composition and immunological response for preventing ST infection in RPS-fed pigs.

RPS is a type II RS that can decrease body weight (BW) in humans and animals. However, the ADG of TRT pigs was not significantly different from that of CON pigs during the RPS feeding period (until 21 dpf), consistent with the finding in our previous study (13). Although *Salmonella* infection in pigs reduces BW and ADG (20), the ADG of TRT pigs was significantly higher than that of CON pigs during the ST infection period. These findings could explain the gut health-promoting effect in RPS-fed pigs because ADG in pigs is strongly related to intestinal morphology (21). Additionally, healthy gut microbiota and its derived SCFAs could prevent the colonization of pathogenic bacteria by decreasing gut mucosal permeability (13, 22). The histopathological lesions in the intestinal organ tissues of TRT pigs were significantly milder than those of CON pigs. Moreover, ST fecal shedding was reduced in RPS-fed pigs (TRT) at 8 dpi. These results suggest improved gut health after the post-weaning diet supplemented with RPS.

The abundance of nine bacterial genera significantly increased in TRT pigs, among which *Blautia* (*P* = 0.0374), *Muribaculum* (*P* = 0.0104)*, Anaerobutyricum* (*P* = 0.0374), and *Anaerocolumna* (*P* = 0.0247) were the main butyrate-producing bacteria (23–26). *Blautia* is considered as a novel potential probiotic due to its ability to produce bacteriocin (sactipeptide and lanthipeptide), inhibit pathogenic bacterial colonization, and regulate inflammatory responses (25). Thus, we speculated that the increased abundance of *Blautia* might have contributed to the mild histopathological lesions and reduced ST colonization in the TRT pigs' intestinal organ tissues. Yuan et al. (27) showed that metabolites from *Muribaculum* could improve gut barrier function and integrity, preventing leakage of inflammatory mediators into the systemic circulation. Moreover, herein, the genus *Anaerobutyricum* and its subtaxon *A. hallii* (*P* = 0.0374) increased more in the TRT group than in the CON group. *A. hallii* is a potential next-generation probiotic bacterium because of its capacity to produce propionate and butyrate (28). Additionally, the four main acetate-producing bacteria in TRT, *Intestinibacter* (*P* = 0.0250)*, Anaerocolumna* (*P* = 0.0247)*, Gracilibacter* (*P* = 0.0374), and *Collinsella* (*P* = 0.0250), were more prevalent in the TRT group than in the CON group. *Anaerocolumna* reportedly decomposes cellulose, oligosaccharides, polysaccharides, and organic acids into energy sources (29). Moreover, *Gracilibacter* can degrade glucose, and *Intestinibacter* is involved in mucin consumption by degrading fucose (30). Lastly, *Collinsella* is significantly and positively correlated with most bile acids and is related to lipid metabolism (31). Conversely, the abundance of only two genera, *Harryflintia* (*P* = 0.0463) and *Solobacterium* (*P* = 0.0278) increased in the CON group, which can only produce acetate (32, 33). Lawhon et al. (34) reported that unbalanced SCFA ratio (e.g., high acetate and low butyrate/propionate concentration) could cause a more invasive ST infection. Further, *Harryflintia* abundance reduced when mice were fed high concentrations of RPS (0–10%) (35). Although the abundant species *Campylobacter lanienae* (*P* = 0.0222) and *Solobacterium moorei* (*P* = 0.0278) are common in the gastrointestinal tract of pigs, they have emerged as a potential cause of human gastroenteritis (32, 36).

Our previous study showed that feeding 5% RPS resulted in significantly higher concentrations of acetate, butyrate, and total SCFAs in healthy pigs (13). In the present study, however, the three SCFAs in the TRT and CON groups showed no significant difference, despite the increased numbers of butyrate-producing bacteria. ST reportedly uses and decreases microbiota-derived butyrate by altering the gut microbiota composition; consequently, the intestinal epithelium shifts to lactate fermentation (37, 38). In addition, sufficient concentrations of butyrate and propionate reportedly enabled the abrogation of ST-induced gut inflammation by regulating the expression of genes responsible for ST invasion and pathogenesis; moreover, they increased the sensitivity of the pathogens to butyrate-mediated repression of invasion-related gene expression (34, 37). Herein, butyrate consumption by ST might not significantly increase butyrate and propionate concentrations in the gut of TRT pigs; however, this may affect the severity and bacterial colonization results in RS-fed pigs.

In the present study, the mRNA expression of *Reg3*γ (antimicrobial peptide gene) markedly increased in the cecum and colon of the TRT pigs. *Reg3*γ restricts bacterial colonization of the intestinal mucosal surface and maintains spatial segregation between bacteria and intestinal epithelium (39). Therefore, the poor colonization of ST in TRT pigs could result from the enhanced *Reg3*γ expression in the cecum and colon. Furthermore, IL-18 mRNA expression levels and histopathological scores were significantly reduced in TRT pigs' colons. Previous studies have suggested that IL-18 is necessary to initiate mucosal inflammation (40, 41). Moreover, the upregulation of IL-18 is central to the pathogenesis of tissue destruction and the severity of gastroenteritis in humans and mice (41, 42). Therefore, the high *Reg3*γ expression and low IL-18 expression in TRT are related to the reduced ST colonization and histopathology results.

In this study, GO analysis revealed that upregulated DEGs were primarily involved in immune and inflammatory responses at 2 dpi. The top five biological processes were the inflammatory response, immune effector process, defense response regulation, adaptive immune response, and response to bacteria. Furthermore, GO enrichment analysis of the upregulated DEG included immune receptor activity, cytokine receptor activity, and cytokine binding. These results are consistent with previous findings that inflammatory features peaked, and inflammatory infiltration significantly increased in the small intestinal tissues of ST-infected piglets at 2 dpi (43, 44). Considering these results together with qPCR results, the GO analysis revealed that ST infection triggered immune responses in the early phase. At 2 dpi, transcript levels of the tight junction proteins claudin and occludin (*CLDND1* and *OCEL1*) in blood increased by 22.33 and 1.21 folds, respectively. However, at 14 dpi, the genes were less expressed in the TRT group than in the CON group. This result suggests that *CLDN and OCLN* might have been expressed earlier, making it unnecessary by 14 dpi. Additionally, at 2 dpi, the cytokine genes related to inflammation, IL-10 subunit alpha and subunit beta (IL10Rα and IL10RB) showed 1.62- and 2.23-fold increases, respectively, and the IL-17 gene (IL17B) showed a 41-fold increase. Moreover, we observed 4.84-, 2.49-, and 1.91-fold increases in IL-18 receptor (IL18R1), binding protein (IL18BP), and IL18 genes, respectively. However, excluding IL-17A in the colon, IL-10 and IL-17A were lower in all tissues examined in this study, and IL-18 was significantly lower in the jejunum and colon of the TRT group than in those of the CON group. *Reg3*γ overexpression reportedly induces high immunosuppression (45). Therefore, the dramatic reduction in cytokine genes in our study might be caused by a significant increase in *Reg3*γ at 14 dpi.

Overall, the results demonstrate that the RS-supplement diet could prevent ST infection in weaned pigs and provide insights into the mechanisms underlying the immune responses of RS-fed pigs against ST infection. However, there are some limitations to providing a general conclusion of using RS feeding as a preventative measure for ST in pigs. First, this study was conducted on a limited number of pigs; therefore, the results may not represent the general pig population. Further studies with larger sample sizes and different breeds of pigs are required to validate these findings. Second, the animal experiment was conducted under controlled conditions; therefore, the results may be difficult to replicate immediately on a pig farm. An application experiment in actual farm units is required in the future.

## 5. Conclusion

The study results suggest that feeding weaned pigs with RPS—a type II RS—could improve gut health and reduce ST infection. The TRT group showed higher ADG during the infection period and milder histopathological lesions in the intestinal organs than the CON group. In addition, TRT pigs exhibited marked reduction in ST shedding compared with that in CON pigs. These results suggested that RPS feeding in weaned pigs could reduce economic losses in farm due to ST infection. The gut microbiota of the TRT group showed an increased abundance of four main butyrate-producing bacteria and four main acetate-producing bacteria. The increased levels of these beneficial bacteria could have contributed to promoting gut health and reducing ST colonization in the TRT group. Moreover, *Reg3*γ expression was markedly increased in the TRT pigs, preventing ST colonization in RPS-fed pigs. Overall, our findings highlight the potential use of RPS as a dietary intervention to improve gut health and reduce *Salmonella* infections in pigs.

## Data availability statement

The datasets presented in this study can be found in online repositories. The names of the repository/repositories and accession number(s) can be found at: https://www.ncbi.nlm.nih.gov/bioproject; PRJNA881483, PRJNA952690.

## Ethics statement

All the experiments were approved by the Animal Ethics Committee of the National Institute of Animal Science, Republic of Korea (Approval No. NIAS 2021-503).

## Author contributions

S-IO made substantial contributions to the conception and design of the work and revised the manuscript prior to the submission. S-WY was responsible for laboratory analyses, data curation, and interpretation of experimental data. S-WY, HL, EK, and S-IO were responsible for animal experiments and investigations. S-WY, Y-HJ, E-YB, AC, YD, T-YH, and S-IO were responsible for data validation and resources. S-WY and S-IO wrote the original draft. All authors read and approved the final manuscript.
